# CH_4_, C_2_H_6_, and C_2_H_4_ Multi-Gas Sensing Based on Mid-Infrared Spectroscopy and SVM Algorithm

**DOI:** 10.3390/s25051427

**Published:** 2025-02-26

**Authors:** Wenyuan Shao, Yunjiang Jia, Xilian Su, Benlei Zhao, Jiachen Jiang, Limei Gao, Xiaosong Zhu, Yiwei Shi

**Affiliations:** 1 School of Information Science and Technology, Fudan University, Shanghai 200438, China; 2 Zhongshan-Fudan Joint Innovation Center, Zhongshan 528400, China

**Keywords:** multi-gas detection, infrared absorption spectroscopy, SVM

## Abstract

A multi-gas sensing system based on mid-infrared spectral absorption was developed for the detection of CH_4_, C_2_H_6_, and C_2_H_4_. The system utilized a broadband infrared source, a hollow waveguide (HWG) absorption cell, and a tunable Fabry–Pérot (FP) detector. The limits of detection (LODs) of CH_4_, C_2_H_6_, and C_2_H_4_ were 7.33 ppm, 2.13 ppm, and 8.09 ppm, respectively. For multi-gas measurements, the support vector machine (SVM) algorithm model was employed to calculate the concentration of each component. The root mean square error of prediction (RMSEP) values for CH_4_, C_2_H_6_, and C_2_H_4_ were 15.91 ppm (1.26%), 1.64 ppm (0.57%), and 6.95 ppm (0.55%), respectively. The generation of stimulated absorption spectra of mixed gases was realized, and the sample selection of measurement for accurate concentration calculation of each gas was optimized. The system proposed in this work provides a simple, miniaturized, and cost-effective solution for multi-gas sensing.

## 1. Introduction

Methane (CH_4_), ethane (C_2_H_6_), and ethylene (C_2_H_4_) are typical pollution gases emitted during industrial production, transportation, and building renovation. Excessive concentrations of CH_4_, C_2_H_6_, and C_2_H_4_ have been demonstrated to have serious impacts on air quality, climate change, and human health [1,2,3,4]. CH_4_ and C_2_H_6_ are the primary components of oil and natural gas, as well as the predominant volatiles produced by the combustion of fossil fuels [5]. Emissions of CH_4_ and C_2_H_6_ contribute to the greenhouse effect and climate change [6,7]. C_2_H_4_, a byproduct of chemical production and motor vehicle exhaust, has been identified as a carcinogen when exposed to high concentrations over an extended period [8]. Consequently, monitoring the emission concentrations of CH_4_, C_2_H_6_, and C_2_H_4_ is of special importance for preventing environmental pollution and protecting human health. In recent years, spectroscopic methods have undergone significant advancement and become increasingly prevalent in the domain of multi-gas monitoring. Fiber-enhanced Raman spectroscopy (FERS) and infrared absorption spectroscopy (IR) are commonly used spectroscopic methods for multi-gas detection.

FRES gas sensors are based on Raman scattering, i.e., inelastic light scattering at the vibrational–rotational or rotational energy level of molecules. Each gas can be identified by its ‘molecular fingerprint’, i.e., its unique vibrational or rotational peak pattern. FERS has the advantage of high accuracy, sensitivity, and resolution. However, because the signal is weak due to the small Raman scattering cross-section, a variety of sophisticated enhancement techniques must be employed to amplify the Raman signal. The use of a high-power laser light source and fiber optic sensing techniques is a common solution [9,10]. In 2020, Andreas Kneb et al. implemented a dual gas sensor with detection limits of 3 ppm and 49 ppm for CH_4_ and H_2_, respectively, using a laser light source and a hollow-core anti-resonant fiber (HC-ARF) absorption cell [11]. In 2022, Bai et al. used a laser light source to design a Raman gas sensor based on HC-ARF for the simultaneous measurement of CH_4_, C_2_H_6_, C_2_H_4_, acetylene (C_2_H_2_), hydrogen (H_2_), and carbon monoxide (CO), with detection limits of 1.2 ppm, 2.9 ppm, and 1.6 ppm for CH_4_, C_2_H_6_, and C_2_H_4_, respectively [12]. Excessive laser intensity usually excites a large number of fluorescence signals during practical measurements, resulting in Raman scattering signals being covered by fluorescence. In 2023, Wan et al. proposed a fluorescence noise elimination (FNE) technique that can effectively eliminate horizontal and vertical spatial noise, and the multi-gas optical sensor based on this technique had detection limits of 1.5 ppm for CH_4_ and 4.8 ppm for C_2_H_4_ [13]. These complex signal enhancement techniques result in complex, bulky, and costly FERS sensing systems, which are not conducive to miniaturization, integration, portability, and commercialization.

IR gas sensors are based on the theory of absorption spectroscopy of gas molecules. Based on the ‘fingerprint’ property of gases in the infrared band, the gas absorbs the corresponding infrared radiation, and the detector produces a thermal change, which is converted into an electrical signal to measure the gas concentration. Unlike FERS, IR gas sensors have a variety of options for light sources and detectors. The use of non-dispersive broadband light sources and pyroelectric detectors is conducive to system miniaturization, integration, and commercialization due to their low cost and small size. In 2020, Tan et al. developed a multi-gas sensing system, comprising a broadband light source and multiple narrowband pyroelectric detectors, with a detection limit of 63 ppm for CH_4_ [14]. In 2020, Liu et al. designed a multi-gas detection sensor composed of a broadband light source and six dual-channel infrared detectors for simultaneous detection of CO, CO_2_, CH_4_, formaldehyde (H_2_CO), ammonia (NH_3_), and nitric oxide (NO) [15]. The detection limit of CH_4_ was 500 ppm, with a detection accuracy of ±0.05%. In 2024, Li et al. designed a greenhouse gas detection sensor consisting of a broadband light source and a four-channel detector [16]. This sensor was used to detect the concentrations of CH_4_, nitrous oxide (N_2_O), and carbon dioxide (CO_2_). The full-scale error of the sensor was found to vary by less than 0.81%, the repeatability error was less than 0.39%, and the stability was less than 0.61%. However, for multi-gas detection, superposition of gas absorption in the infrared spectrum leads to crosstalk, significantly impacting detection accuracy [17]. To address the challenge, machine learning algorithms have been employed to eliminate interference between gases, enhancing the performance of the system [18,19,20].

In this work, a portable mid-infrared spectroscopic gas sensing system was established. It consists of a broadband light source, a tunable Fabry–Pérot (FP) filter detector, and a hollow waveguide (HWG) absorption cell. The emission spectrum (2–14 μm) of the broadband light source encompasses the mid-infrared absorption bands of CH_4_ (3.2–3.6 μm), C_2_H_6_ (3.3–3.6 μm), and C_2_H_4_ (3.1–3.5 μm). The pyroelectric detector is capable of performing wavelength scan detection in the band of 3.1–4.4 μm. The light source and detector are both characterized by their low cost and small size, which facilitates system miniaturization, integration, and commercialization. The HWG functions as both a transmission medium for mid-infrared light and a gas absorption cell [21,22]. The HWG is flexible and of low loss property owing to the inner coatings of Ag and AgI (Ag/AgI) optical films. It can be directly coupled to the light source and detector, enhancing the system’s engineering stability [23]. The system involved the detection of single-component concentrations of CH_4_, C_2_H_6_, and C_2_H_4_, as well as the detection of three-component mixtures. To address the challenge of superposition in characteristic absorption spectra of multiple gases, a support vector machine (SVM) algorithm was employed for concentration calculation. In an effort to reduce expenses and enhance efficiency, this work proposes the utilization of a limited number of measured spectra of mixed gases to generate a substantial quantity of simulated spectra, subsequently employing the simulated spectra for model training.

## 2. Principle and System

### 2.1. Sensing System

**Figure 1:**
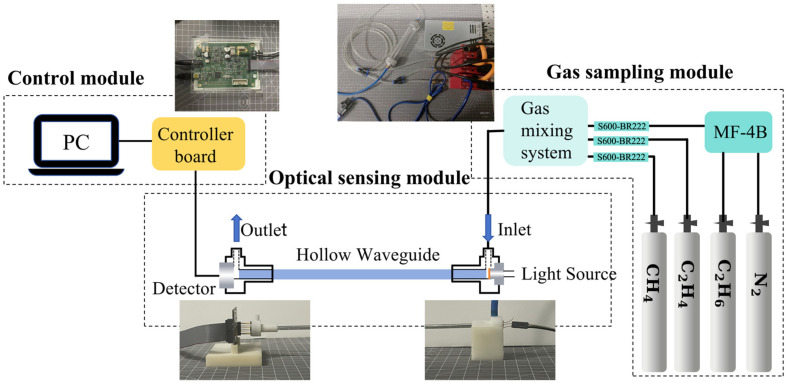
Schematic diagram of the established sensing system.

A multi-gas sensing system was established as shown in Figure 1. The system was composed of three modules: the gas sampling module, the optical sensing module, and the control module.

**Figure 2:**
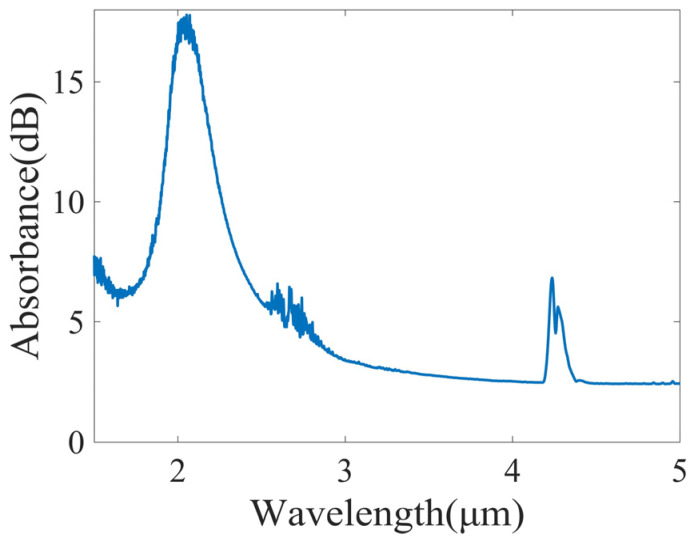
Measured loss spectrum of the HWG by using a Fourier transform infrared spectrometer. The inner diameter of the HWG is 4 mm, with a length of 110 cm.

The optical sensing module mainly consisted of an infrared light source, a detector, and a HWG absorption cell. The light source was a commercial mid-infrared broadband light source (Axetris, EMIRS200, Sarnen, Switzerland). It emits light at wavelengths of 2–14 μm, covering the characteristic absorption bands of CH_4_ (3.2–3.6 μm), C_2_H_6_ (3.3–3.6 μm), and C_2_H_4_ (3.1–3.5 μm). The detector (Infratech, LFP-3144(C)-337, Dresden, Germany) was a pyroelectric detector with a tunable FP filter. The detection wavelength range is 3.1–4.4 μm, and the wavelength resolution is approximately 68–85 nm. The HWG functions as both a transmission medium for mid-infrared light and a gas absorption cell. It has the advantages of low loss, small size, flexibility, and fast response. This system used an Ag/AgI HWG with an inner diameter of 4 mm and a length of 110 cm. Figure 2 presents the measured loss spectrum of the HWG in the mid-infrared band. As can be seen, the loss of the HWG in the detection band of 3.1–4.4 μm is low. The loss peak at 4.3 μm in the loss spectrum is the intrinsic absorption of CO_2_ in air, and the peak at 2 μm is the interference peak of the AgI film. The mid-infrared light emitted by the broadband light source was directly coupled into the HWG through a 3D-printed jointer. The mid-infrared light was transmitted in the HWG and absorbed by the gas. Then it was directly coupled through another jointer and detected by the FP detector.

The control module was composed of a computer and controller boards (Infratech, FPI-EvaIKit, Dresden, Germany). The controller board was linked to the computer and a secondary controller board that controls the light source and detector. The computer was responsible for regulating the drive current of the light source and configuring the parameters of the FP detector. It received the signal detected by the detector. The operational wavelength band of the detector configured in this work is 3.1–4.2 μm, with a scanning step of 0.02 μm. A single scan cost 43 s, encompassing a total of 56 data points.

The gas sampling module consisted of a multi-component dynamic gas dispenser (National Institute of Metrology, MF-4B, Beijing, China), three mass flow controllers (HORIBA, S600-BR222, Shanghai, China), and a mixing tube. The output gas of each mass flow controller was connected to the mixing tube and flowed into the HWG. The computer controlled the output gas flow rate of each mass flow controller. This gas sampling system was capable of receiving four types of gases, one of which is pure nitrogen (N_2_). Consequently, it was capable of achieving a mixed configuration of three gases. The standard gases utilized in this work were high-purity N_2_ gas (N_2_ ≥ 99.999%, H_2_O ≤ 3 ppm, CO_2_ ≤ 1 ppm, Chemical Center of Fudan University, Shanghai, China), as well as standard 3000 ppm CH_4_ gas, standard 3000 ppm C_2_H_6_ gas, and standard 3000 ppm C_2_H_4_ gas (Air Liquide, Shanghai, China).

### 2.2. Principle

#### 2.2.1. Lambert–Beer’s Law

The basic principle of IR gas sensors is Lambert–Beer’s law, written as follows:

(1) $$A=ln\frac{I_{0}(\nu )}{I_{t}(\nu )}=\sigma (\nu )\cdot L\cdot C$$

where A is the absorbance (dB), I_0_ is the incident light intensity, I_t_ is the transmitted light intensity, σ(ν) is the absorption cross-section of the gas (cm^2^/molecule), L is the optical path length (cm), and C is the measured gas concentration (molecule/cm^3^).

According to Lambert–Beer’s law, the absorbance is linearly related to the concentration of the gas when light scattering and the effects of the system itself are excluded, and the optical path length is fixed. Therefore, the absorbance spectrum can be utilized to directly calculate the concentration of the gas.

#### 2.2.2. Support Vector Machine Regression

**Figure 3:**
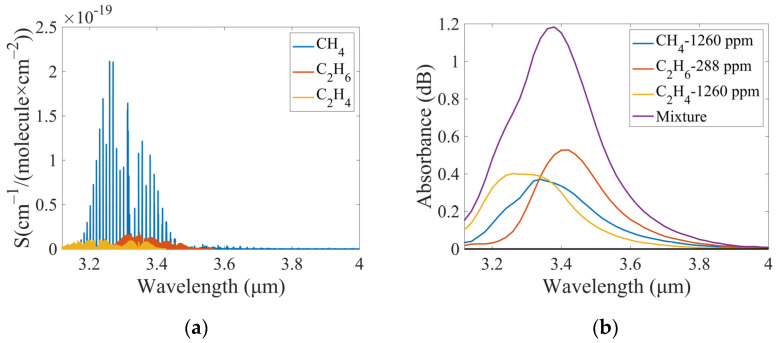
(**a**) Absorption line strength of CH_4_, C_2_H_6_, and C_2_H_4_ from HITRAN. (**b**) Measured absorption spectra of CH_4_, C_2_H_6_, C_2_H_4,_ and their mixture by the established sensing system.

The sensing system established in this work is capable of detecting multiple gases within a specific spectral range in a simultaneous manner. However, the measured spectrum is a superposition of multiple gas absorption spectra due to the low wavelength resolution of the FP detector, especially for gases with characteristic absorption bands that are close to or overlap. Therefore, it is not possible to directly calculate the concentration of each gas from the superimposed absorption spectrum of the mixed gases. Figure 3a shows the absorption line strength of CH_4_, C_2_H_6_, and C_2_H_4_ from the HITRAN database. Figure 3b presents the measured spectra of CH_4_, C_2_H_6_, and C_2_H_4_ separately, as well as their mixture, utilizing the sensing system established in this work. It is evident that the characteristic absorption bands of the three hydrocarbon gases overlap to a significant extent. To address the challenge, a nonlinear mapping algorithm, known as the SVM, was employed to construct a nonlinear model. This model utilizes the absorption spectra of the mixed gases to calculate the concentration of each of the three gases.

SVM has the advantages of being versatile, robust, and computationally simple. With training data H = {(x_i_,y_i_)}, i = 1, 2…n, where x_i_ is the input quantity and y_i_ is the corresponding output quantity, there is a fitting function, written as follows:

(2) $$f(x)=\langle \omega ,x\rangle +b$$

where ω is a hyperplane in space and b is a bias.

In a nonlinear problem, the training data, x_i_, are preprocessed by mapping Φ: X → F onto a high-dimensional feature space F. At this point, the expression of f(x) is as follows:

(3) $$f(x)=\langle \omega ,\Phi (x)\rangle +b$$

According to the principle of structural risk minimization, this optimization problem can be formulated equivalently as follows:

(4) $$min\frac{1}{2}{∥w∥}^{2}+c\sum_{i=1}^{n}\left(\xi_{i}+\xi_{i}^{*}\right)\;s.t.\left\{\begin{array}{c} y_{i}-\langle \omega ,\Phi (x_{i})\rangle -b\leq \varepsilon +\xi_{i}\\ \langle \omega ,\Phi (x_{i})\rangle -b-y_{i}\leq \varepsilon +\xi_{i}^{*}\\ \xi_{i}+\xi_{i}^{*}\geq 0(i=1,2,\cdots ,n) \end{array}\right.$$

where n is the number of samples, c is the penalty factor, ξ is the relaxation factor, and ε is the insensitivity parameter, which is used to reflect the model’s tolerance of errors.

By introducing the Lagrange multiplier, the dual problem of the above problem can be obtained as follows:

(5) $$max[-\frac{1}{2}\sum_{i,j=1}^{n}\left(\alpha_{i}-\alpha_{i}^{*}\right)(\alpha_{j}-\alpha_{j}^{*})\langle \Phi (x_{i}),\Phi (x_{j})\rangle -\sum_{i=1}^{n}\alpha_{i}(\varepsilon -y_{i})-\sum_{i=1}^{n}\alpha_{i}^{*}(\varepsilon +y_{i})]\;s.t.\left\{\begin{array}{c} \sum (\alpha_{i}-\alpha_{i}^{*})=0\\ 0\leq \alpha_{i},\alpha_{i}^{*}\leq c(i=1,2,\cdots ,n) \end{array}\right.$$

The above optimization problem is solved using quadratic programming in optimization theory to obtain the parameters α_i_ and α_i_*.

The final calculated predicted output is written as follows:

(6) $$f(x)=\sum_{i=1}^{n}(\alpha_{i}-\alpha_{i}^{*})\langle \Phi (x_{i}),\Phi (x)\rangle +b$$

A kernel function k that satisfies Mercer’s theory is introduced to map the input space to a high-dimensional feature space that is separable or approximately separable. An SVM-based feature model is obtained by quadratic optimization in the feature space, and the fitting function can be expressed as follows:

(7) $$f\left(x\right)=\sum_{i=1}^{n}\left(\alpha_{i}-\alpha_{i}^{*}\right)K(x,x_{i})+b$$

Since the quantitative analysis of mixed gases involved in this paper is a nonlinear modeling problem, the radial basis function (RBF) kernel function, which has the best nonlinear fitting effect, is selected as the kernel function for SVM modeling. Its expression is written as follows:

(8) $$K\left(x,x_{i}\right)=exp\left(g{\left\|x-x_{i}\right\|}^{2}\right)$$

where g is a parameter of the kernel function and g > 0.

**Figure 4:**
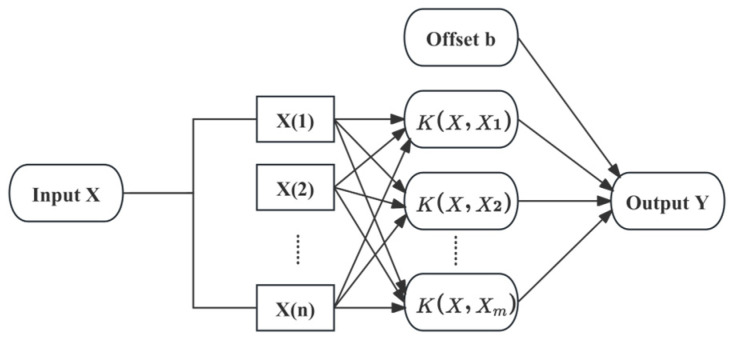
Topology of SVM.

The topological structure of the SVM algorithm is shown in Figure 4. Where X is the input variable, K is the kernel function, Y is the output, and b is the bias.

#### 2.2.3. Evaluation Parameters for SVM

The root mean square error of calibration (RMSEC) is used to evaluate the fitting accuracy of the SVM model.

(9) $$RMSEC=\sqrt{\frac{\sum_{i=1}^{n}{\left(y_{i}-y_{i}^{\prime }\right)}^{2}}{n}}$$

where n is the number of samples used for training, y_i_ is the actual value of the ith sample, and y_i_’ is the correction value of the ith sample.

The smaller RMSEC value means the higher fitting accuracy of the model.

The root mean square error of prediction (RMSEP) is used to evaluate the prediction accuracy of the SVM model.

(10) $$RMSEP=\sqrt{\frac{\sum_{i=1}^{m}{\left(y_{i}-y_{i}^{\prime }\right)}^{2}}{m}}$$

where n is the number of samples used for prediction, y_i_ is the actual value of the ith sample, and y_i_’ is the predicted value of the ith sample.

The smaller RMSEP value means the higher prediction accuracy of the model.

## 3. Results and Discussion

### 3.1. Measurement and Analysis of Single-Component Gas

To evaluate the detection performance of the system, measurements were firstly carried out with single-component gas.

First, the concentration of CH_4_ was controlled by the gas sampling module, and the range of variation was 0–360 ppm with a variation interval of 60 ppm. The absorbance spectrum of each gas concentration was measured five times and averaged. Baseline calibration was performed using the method described in Equation (11).

(11) $$A_{c}=A-\frac{\sum_{i=1}^{k}A_{i}}{k}$$

where A_i_ is the absorbance value of the selected reference band for spectral baseline calibration, which is usually a band without gas absorption. In this paper, 4.0–4.2 μm is selected as the reference band. k is the number of data points in the reference band, A is the absorbance value before calibration and A_C_ is the absorbance value after calibration.

**Figure 5:**
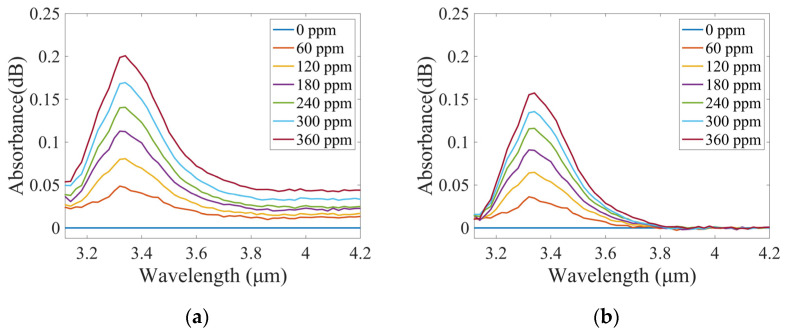
(**a**) CH_4_ absorption spectra before baseline calibration; (**b**) CH_4_ absorption spectra after baseline calibration.

A comparison of the gas absorption spectra before and after baseline calibration is shown in Figure 5. It can be seen that this method can solve the baseline drift problem very well.

**Figure 6:**
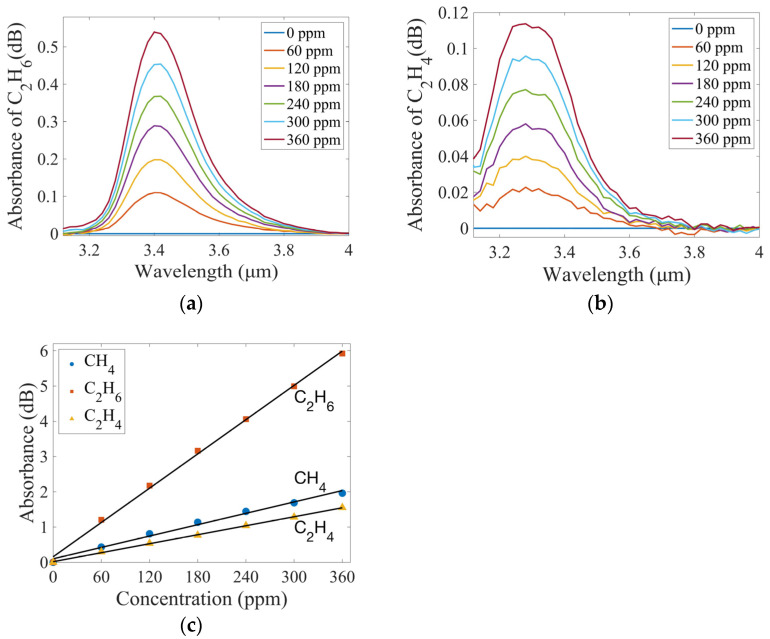
Measured absorption spectra of (**a**) C_2_H_6_, and (**b**) C_2_H_4_ with different concentrations; (**c**) Experimental data and fitting curve of CH_4_, C_2_H_6_, and C_2_H_4_ concentration versus absorbance area.

In this work, the absorption peak summation method was used to calculate the absorbance of the gas. After obtaining the absorption spectrum of CH_4_ at a given concentration, the absorbance of CH_4_ at each wavelength point in the range of 3.18–3.56 μm was summed to obtain the absorbance of the gas at that concentration. The gas concentration was fitted to the corresponding absorbance value using a polynomial and the least squares method was used to determine the slope and intercept. The relationship between gas concentration and absorbance is shown in Figure 6c, which is linear. Equation (12) is the fitting equation, and the correlation coefficient R-squared is 0.9904, written as follows:

(12) $$A_{{CH}_{4}}=0.0054\times c_{{CH}_{4}}+0.0998$$

By using the same measurement and analysis methods, the concentration changes of C_2_H_6_ and C_2_H_4_ were configured. The absorption spectra of C_2_H_6_ and C_2_H_4_ are shown in Figure 6a,b. Based on the absorption characteristics of each gas, the wavelength range where the absorbance of C_2_H_6_ is summed was 3.3–3.6 μm, and the wavelength range of C_2_H_4_ was 3.14–3.48 μm. The linear relationship between gas concentration and absorbance is also shown in Figure 6c. The fitting equations are given in Equations (13) and (14). The R-squared values are 0.9981 and 0.9993, respectively.

(13) $$A_{{C_{2}H}_{6}}=0.0162\times c_{{C_{2}H}_{6}}+0.1572$$

(14) $$A_{{C_{2}H}_{4}}=0.0042\times c_{{C_{2}H}_{4}}+0.0179$$

Limit of detection (LOD) is used to evaluate the performance of a gas sensing system.

(15) $$LOD=\frac{3\sigma }{s}$$

where σ is the standard deviation of the measured spectra of the blank signal (pure N_2_) and s is the sensitivity, which is the slope of the curve fitted to the absorbance and gas concentration.

The standard deviation was calculated by measuring 10 blank signals with pure N_2_. Then, combined with the sensitivity of the system for the three gases in Figure 6c, the LODs of the system for CH_4_, C_2_H_6_, and C_2_H_4_ were calculated to be 7.33 ppm, 2.13 ppm, and 8.09 ppm, respectively. Linear regression analysis shows that the R-squared values for CH_4_, C_2_H_6_, and C_2_H_4_ were 0.9904, 0.9981, and 0.9993, respectively. This indicates that the sensing system established in this work has high linear performance and can detect gases at ppm level.

The related research on multi-gas detection based on FRES and IR in recent years is summarized in Table 1. Compared with the FRES system, the proposed system has the advantages of low cost, small size, simple structure, and easy maintenance. Compared with other IR systems that use broadband light sources and pyroelectric detectors, the proposed system has higher detection accuracy. The proposed system achieves a compromise between cost and performance.

**Table 1:** Summary of related research on multi-gas detection based on FERS and IR.

Method	Target Gas	Detection Limit/ Detection Error	Reference	Year
FERS	CH_4_	3 ppm	[11]	2020
	H_2_	49 ppm		
FERS	CH_4_	1.2 ppm	[12]	2022
	C_2_H_6_	2.9 ppm		
	C_2_H_4_	1.6 ppm		
	C_2_H_2_	2.7 ppm		
	H_2_	13.8 ppm		
	CO	16.7 ppm		
FERS	CH_4_	1.5 ppm	[13]	2023
	C_2_H_4_	4.8 ppm		
	C_2_H_2_	3.1 ppm		
	CO_2_	9.4 ppm		
	CO	1 ppm		
IR	CH_4_	500 ppm	[14]	2020
	CO_2_	100 ppm		
	CO	500 ppm		
IR	CH_4_	63 ppm	[15]	2020
	CO_2_	2 ppm		
	CO	11 ppm		
IR	CH_4_	−0.34%~−0.77%	[16]	2024
	CO_2_	−0.40%~−0.26%		
	N_2_O	−0.81%~−0.32%		
IR	CH_4_	7.33 ppm	This work	-
	C_2_H_6_	2.13 ppm		
	C_2_H_4_	8.09 ppm		

### 3.2. Measurement and Analysis of Mixed Gases

**Figure 7:**
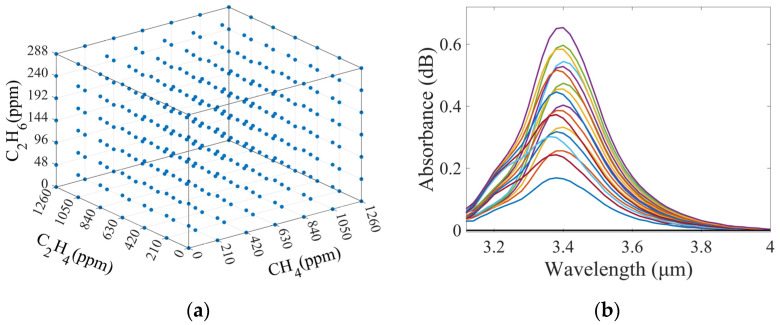
(**a**) Gas concentration distribution of 343 gas mixture samples; (**b**) Typical measured absorption spectra of 343 gas mixture samples. Different color lines represent absorption spectra for different concentration combinations of gas mixture samples.

In the experiment, the flow rate of three standard gases was adjusted by the gas sampling system, and high-purity N_2_ gas was used for dilution to prepare gas mixture samples with different concentration combinations. The concentration range of CH_4_ was 0–1260 ppm, with an interval of 210 ppm, for a total of seven concentration data points. The concentration range of C_2_H_6_ was 0–288 ppm, with an interval of 48 ppm, for a total of seven concentration data points. The concentration range of C_2_H_4_ was 0–1260 ppm, with a concentration interval of 210 ppm, for a total of seven concentration data points. The concentrations of the three gases were mixed and configured to provide a total of 7 × 7 × 7 = 343 different concentration combinations of CH_4_, C_2_H_6_, and C_2_H_4_ gas mixture samples. The concentration configurations of the 343 samples are shown in Figure 7a.

Each concentration combination was measured three times to obtain the absorption spectra. The data were then baseline calibrated and averaged. Typical measured absorption spectra are shown in Figure 7b. Due to the low wavelength resolution of the FP detector used in the system, samples containing a mixture of CH_4_, C_2_H_6_, and C_2_H_4_ gases exhibit a superposition of absorption spectra in the measured spectra. Therefore, it is difficult to directly extract the spectral information of each component gas from the measured spectra to calculate the concentration of each gas. Hence, the SVM algorithm was used to calculate the concentration of multi-component gases with such spectral superposition.

The sensing system is capable of detecting a wavelength range of 3.1–4.2 μm, with a step of 0.02 μm and a total of 56 data points scanned. Based on the gas absorption line strength obtained from the HITRAN database (see Figure 3a) and the measured absorption spectra (see Figure 3b), 45 data points in the 3.12–4.0 μm wavelength range were finally selected for SVM model training. After baseline calibration and selection of the spectral wavelength range, the spectral data matrix X and concentration data matrix Y for modeling were formed.

(16) $$X=\left[x_{ij}\right]=\left[\begin{array}{ccc} x_{11} & \cdots & x_{1n}\\ \vdots & \ddots & \vdots \\ x_{m1} & \cdots & x_{mn} \end{array}\right]Y=\left[y_{ij}\right]=\left[\begin{array}{ccc} y_{11} & \cdots & y_{1m}\\ \vdots & \ddots & \vdots \\ y_{p1} & \cdots & y_{pm} \end{array}\right]$$

where m is the number of samples, n is the number of data points, p is the number of components, x_ij_ is the absorbance value of the jth sample at the ith data point, and y_ij_ is the concentration value of the ith component in the jth sample.

After standardizing the matrix X and Y, they can be used to train the SVM model. The penalty factor c and the kernel function parameter g were initialized. If the former is relatively large and the latter is relatively small, the established model will be more complex and tend to be overfitting; if the former is relatively small and the latter is relatively large, the established model will be simpler and tend to be underfitting. The optimal penalty factor c and kernel function parameter g were determined using 5-fold cross-validation, and the model was trained using the optimal parameters.

In order to evaluate the performance of the established SVM model, the leave-one-out cross-validation method was used to train and evaluate the SVM model using 343 sets of measured spectra. The RMSEC and RMSEP values of CH_4_, C_2_H_6_, and C_2_H_4_ are shown in Table 2.

**Figure 8:**
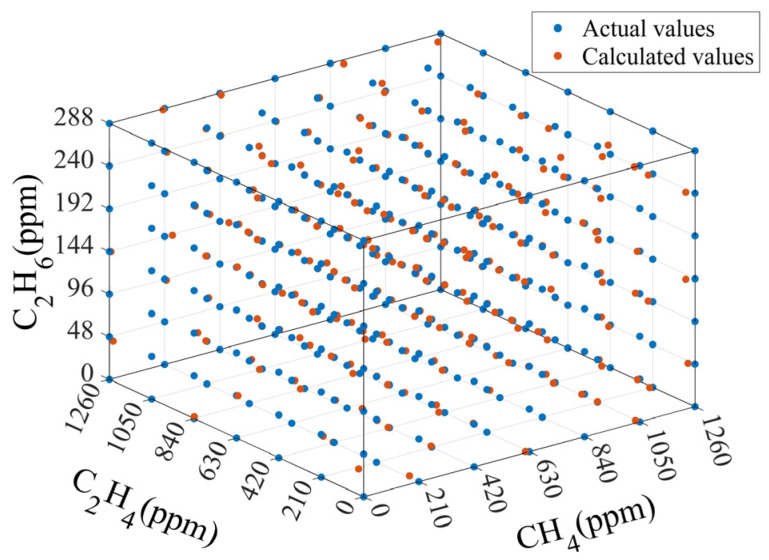
Actual concentrations of CH_4_, C_2_H_6_, and C_2_H_4_ and calculated ones using SVM.

The RMSEC values for CH_4_, C_2_H_6_, and C_2_H_4_ are 9.97 ppm (0.79%), 0.36 ppm (0.13%), and 1.47 ppm (0.12%), respectively. None of them exceeds 0.79% of the full scale of each component. These values demonstrate that the SVM algorithm is capable of performing model fitting with a high degree of accuracy. The RMSEP values for CH_4_, C_2_H_6_, and C_2_H_4_ are 15.91 ppm (1.26%), 1.64 ppm (0.57%), and 6.95 ppm (0.55%), respectively. None of them exceeds 1.26% of the full scale of each component. This indicates that the concentration values calculated by the SVM algorithm have very little error compared to the actual values. A comparison of the actual concentration values of each sample with the calculated values is shown in Figure 8. The results demonstrate the SVM algorithm’s effectiveness in addressing the spectral superposition problem of the three gases.

The RMSEP value of CH_4_ is the worst, which may be because the characteristic absorption peak of CH_4_ is most severely affected by infrared spectral overlap in the 3.1–4.0 μm band. In addition, as SVM can only output one dimension at a time, three models are required to calculate the concentration of these three gases. When modeling, different parameter choices can result in different RMSEP values. Therefore, meticulous parameter tuning is required.

### 3.3. Simulation-Assisted Training

#### 3.3.1. Generation of Simulated Spectra

The SVM algorithm has been demonstrated to be an effective solution for the quantitative analysis of mixed gases with spectral superposition of CH_4_, C_2_H_6_, and C_2_H_4_. However, a large number of measured spectra were required to train the model. In order to improve measurement efficiency, this work proposes a method of generating a large number of simulated spectra from a small number of measured spectra. Then the model can be trained by using a large amount of generated spectra.

In the first step of the process, the spectrum of a single gas is generated. The absorption spectrum is calculated according to Equation (1). The optical path length is the length of the HWG. The absorption line strength of CH_4_ can be obtained from the HITRAN database. And the absorption cross-section can be calculated by converting the absorption line strength. At least one measured absorption spectrum with a known concentration is necessary to derive the parameters such as the divergence angle of the light source and the FP filter properties required for the simulation. Then, a group of simulated CH_4_ spectra at different concentrations can be obtained by changing the gas concentration in the simulation. To improve the accuracy of simulations, a correction factor δ is introduced to correct the error between the simulated and measured spectra of CH_4_ for a group of uniformly distributed concentrations, as shown in Equation (18). Therefore, a minimum of two different concentrations within the concentration measurement range have to be measured in order to simulate CH_4_ absorption spectra for a group of uniformly distributed concentrations.

(17) $$\delta (\lambda )=(\frac{S_{i}(\lambda )-M_{i}(\lambda )}{S_{i}(\lambda )}-\frac{S_{j}(\lambda )-M_{j}(\lambda )}{S_{j}(\lambda )})/(i-j)$$

(18) $$\delta_{k}\left(\lambda \right)=\frac{S_{j}\left(\lambda \right)-M_{j}\left(\lambda \right)}{S_{j}\left(\lambda \right)}-\left(j-k\right)*\delta \left(\lambda \right)$$

(19) $$A_{k}\left(\lambda \right)=S_{k}\left(\lambda \right)\times \left(1+\delta_{k}\left(\lambda \right)\right)$$

where M_i_ and M_j_ are the measured spectra at the ith and jth concentrations, S_i_ and S_j_ are the pre-correction simulated spectra at the ith and jth concentrations, A_k_ is the post-correction simulated spectra at the kth concentration, 2 ≤ i < j ≤ 7, 2 ≤ k ≤ 7.

**Figure 9:**
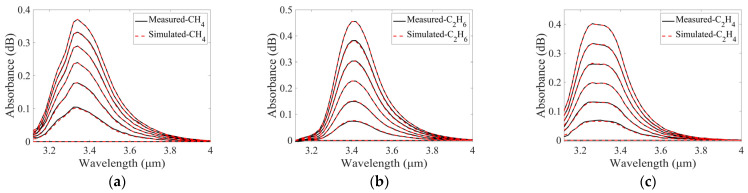
Comparisons of measured and stimulated absorption spectra of (**a**) CH_4_, (**b**) C_2_H_6_, and (**c**) C_2_H_4_ at different concentrations.

Spectra of CH_4_ were measured at concentrations of 840 and 1260 ppm. A group of simulated CH_4_ spectra was generated from the measured spectra. The concentration of CH_4_ varied from 0 to 1260 ppm with an interval of 210 ppm. A comparison of the measured and simulated CH_4_ spectra is shown in Figure 9a.

Similar processes were used to obtain the simulated spectra of C_2_H_6_ and C_2_H_4_. The spectra of C_2_H_6_ at concentrations of 192 and 288 ppm and the spectra of C_2_H_4_ at concentrations of 840 and 1260 ppm were measured and used in the simulation. Two groups of spectra were generated, C_2_H_6_ concentration varying from 0 to 288 ppm with an interval of 48 ppm and C_2_H_4_ concentration varying from 0 to 1260 ppm with an interval of 210 ppm. Comparisons between the measured and simulated spectra of C_2_H_6_ and C_2_H_4_ are demonstrated in Figure 9b,c, respectively.

As illustrated in Figure 9, the simulated single-gas absorption spectra of CH_4_, C_2_H_6_, and C_2_H_4_ are in good agreement with the corresponding measured absorption spectra. Subsequently, the simulated spectra of the mixed gases of CH_4_, C_2_H_6_, and C_2_H_4_ can be obtained from the simulated spectra of the single gases.

In the second step of the process, the simulated spectrum of the mixed gases is generated. Theoretical and experimental results have shown that the spectrum of the mixed gases cannot be obtained by simply adding the spectra of each gas due to the non-linear relationship between the absorbance and gas concentration [24]. Equation (20) was used to generate the spectrum of mixed gases and a correction factor δ is introduced to correct the error between the simulated and measured spectrum.

(20) $$A(\lambda )\left\{\begin{array}{c} \;(A_{CH_{4}}(\lambda )+A_{C_{2}H_{6}}(\lambda ))\times (1+\delta_{{CH}_{4}-C_{2}H_{6}}(\lambda ))\\ \;(A_{CH_{4}}(\lambda )+A_{C_{2}H_{4}}(\lambda ))\times (1+\delta_{{CH}_{4}-C_{2}H_{4}}(\lambda ))\\ \;(A_{C_{2}H_{4}}(\lambda )+A_{C_{2}H_{6}}(\lambda ))\times (1+\delta_{{C_{2}H}_{4}-C_{2}H_{6}}(\lambda ))\\ \;(A_{CH_{4}}(\lambda )+A_{C_{2}H_{6}}(\lambda )+A_{C_{2}H_{4}}(\lambda ))\times (1+\delta_{{{{CH}_{4}-C}_{2}H}_{4}-C_{2}H_{6}}(\lambda )) \end{array}\right.$$

**Figure 10:**
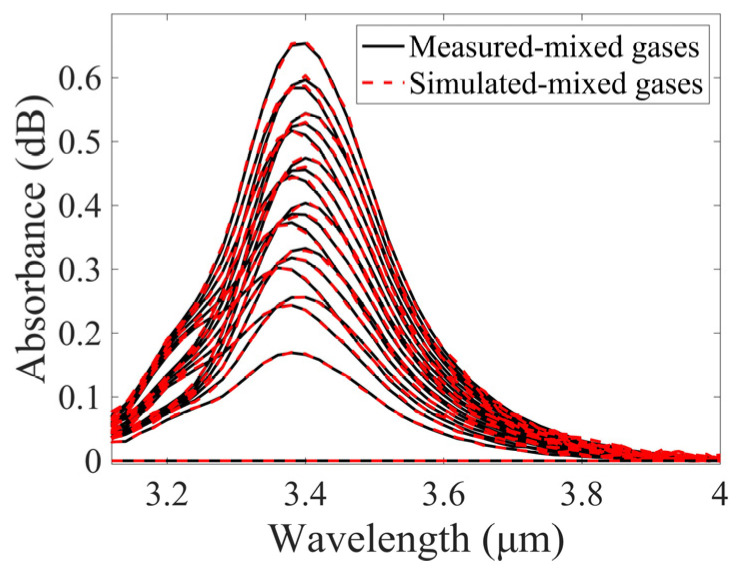
Comparison of measured and stimulated absorption spectra of CH_4_, C_2_H_6_, and C_2_H_4_ mixed gases at different concentrations.

**Figure 11:**
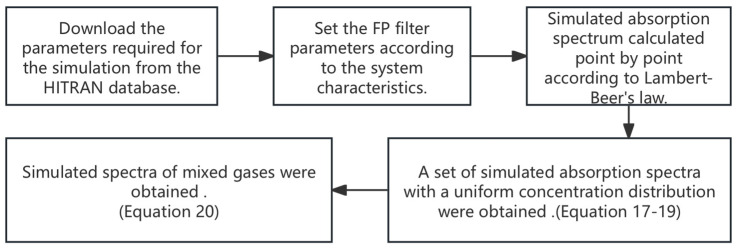
The flowchart for the process of generating simulated spectra.

The value of δ needs be determined based on the measured spectrum of the mixed gases and the corresponding summed simulated spectrum. As illustrated in Figure 10, a comparison of the simulated spectra with the measured spectra is presented. It can be seen that there is a high degree of agreement between the two groups of spectra. This means that a small number of measured spectra can be used to generate a substantial number of simulated spectra for training the SVM model. The flowchart for the process of generating simulated spectra is shown in Figure 11.

#### 3.3.2. Selection of Measured Spectra

Based on a limited number of measured spectra, it is possible to generate 343 sets of simulated spectra corresponding to 343 measured ones to train the SVM model. The 343 sets of simulated spectra were used as the training set, and the 343 sets of measured spectra were used as the test set. The RMSEP values of each component were calculated separately.

Considering the index and calibration of the SVM model, the number of measured spectra and the methods of selection of measured spectra will have an impact on the accuracy of the calculation. To this end, a comparative study was conducted in terms of the number of measured spectra and spectra selection methods. In practice, it prefers fewer measured spectra to lower the cost. Certainly, calculations with high accuracy are expected for more measured spectra.

**Figure 12:**
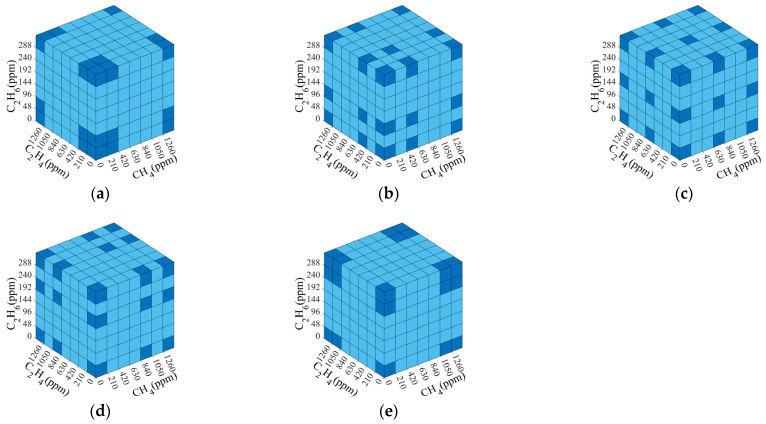
(**a**–**e**) Five typical selection methods for selecting 27 measured spectra. Dark blue squares represent the measured spectra that were selected. Light blue squares represent the measured spectra that were not selected.

There exist many selection methods even if the number of measured spectra is the same. Figure 12 shows five typical selection methods for 27 measured spectra among 343 concentration combinations. Each small square represents a spectrum of a possible concentration combination, and the darker squares are the measured spectra that were selected. The selections were based on the principle that the measured spectra were relatively concentrated or dispersed within the concentration measurement range. Among them, the concentration distribution was relatively concentrated in the selection methods shown in Figure 12a,e, and the concentration distribution was uniformly dispersed in the selection method shown in Figure 12c.

**Figure 13:**
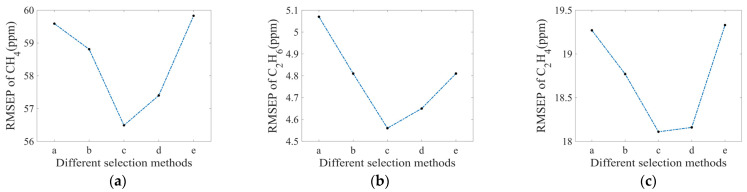
RMSEP values of (**a**) CH_4_, (**b**) C_2_H_6_, and (**c**) C_2_H_4_ using different selection methods with 27 measured spectra.

Figure 13 shows the RMSEP values corresponding to the five selection methods for CH_4_, C_2_H_6_, and C_2_H_4_. It can be seen that the RMSEP values were larger when methods a and e were selected, which the concentration distribution of the spectra was relatively concentrated, indicating larger errors between the calculated and actual concentrations. While selection method c with the most uniformly dispersed concentration distribution, resulted in the smallest RMSEP value, suggesting minimal error between the calculated and actual concentrations. This analysis shows the importance of the spectra selection method, indicating that the selection of uniformly distributed spectra in the combinations is good for a high-accuracy calculation of the algorithm.

**Figure 14:**
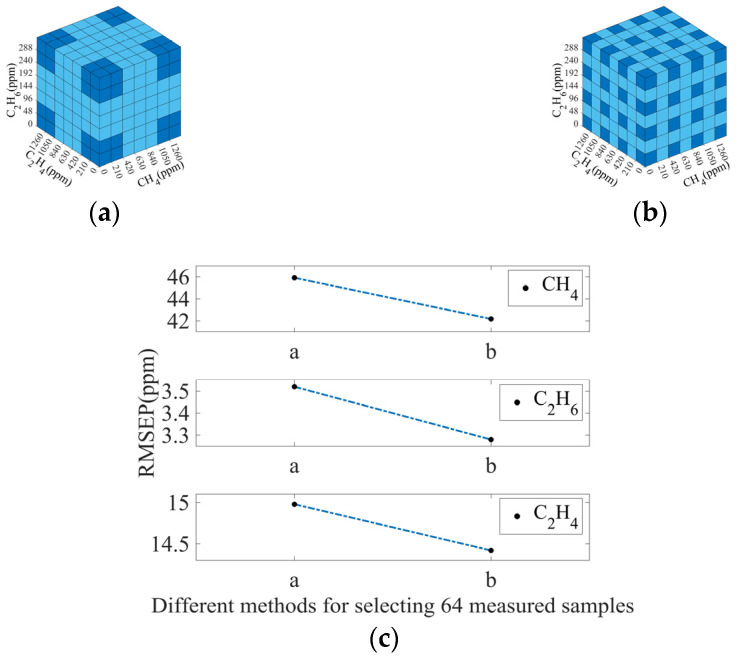
(**a**,**b**) Two typical selection methods for selecting 64 measured spectra. Dark blue squares represent the measured spectra that were selected. Light blue squares represent the measured spectra that were not selected; (**c**) RMSEP values of CH_4_, C_2_H_6_, and C_2_H_4_ using different selection methods for selecting 64 measured spectra.

Figure 14c shows the results with 64 measured spectra. It is the same that measured spectra with uniformly dispersed concentrations output high calculation accuracy.

**Figure 15:**
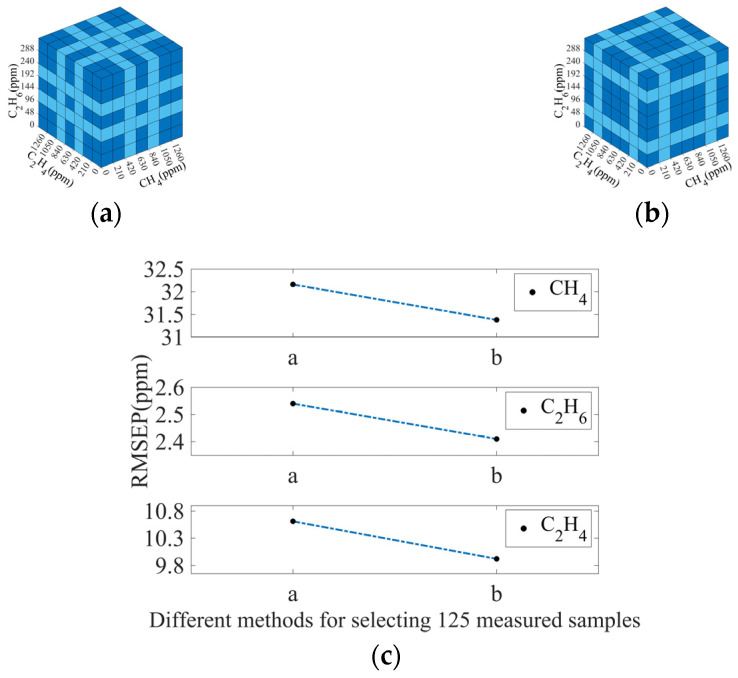
(**a**,**b**) Two typical selection methods for selecting 125 measured spectra. Dark blue squares represent the measured spectra that were selected. Light blue squares represent the measured spectra that were not selected; (**c**) RMSEP values of CH_4_, C_2_H_6_, and C_2_H_4_ using different selection methods for selecting 125 measured spectra.

A comparison was also made when 125 measured spectra were used. In this case, the method in Figure 15a dropped more spectra with concentrations that appear in the median of the measurement range, while the method in Figure 15b retained more spectra with concentrations in the area. As shown in Figure 15c, method b has better accuracy than that of method a. This result indicates that retaining more samples with intermediate values in the concentration distribution can improve the accuracy of the algorithm.

**Figure 16:**
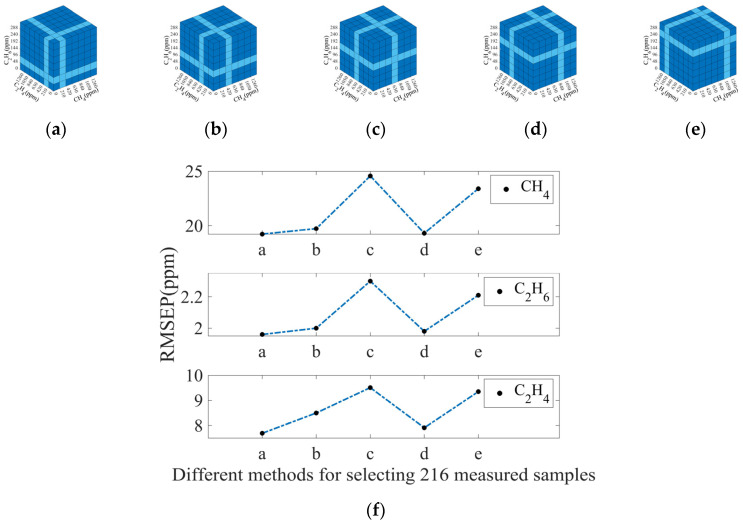
(**a**–**e**) Five typical selection methods for selecting 216 measured spectra. Dark blue squares represent the measured spectra that were selected. Light blue squares represent the measured spectra that were not selected; (**f**) RMSEP values of CH_4_, C_2_H_6_, and C_2_H_4_ using different selection methods for selecting 216 measured spectra.

Considering the selection of 216 measured spectra, five typical selection methods were considered. Among them, method c dropped the middle value of the concentration measurement range compared to the other methods. As shown in Figure 16f, the RMSEP values for CH_4_, C_2_H_6_ and C_2_H_4_ were the largest when method c was selected. It further indicates the importance of keeping spectra as much as possible in the middle of the concentration measurement range.

**Figure 17:**
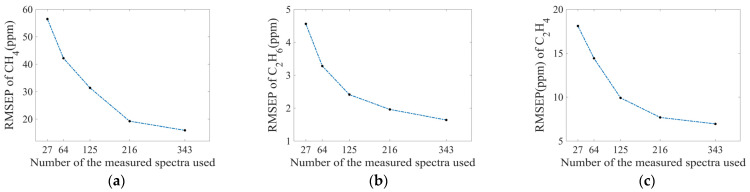
The minimum RMSEP value of (**a**) CH_4_, (**b**) C_2_H_6_, and (**c**) C_2_H_4_ obtained by training simulated spectra data generated from samples with different measured quantities.

Finally, the influence of the number of measured spectra on calculation accuracy was investigated. Figure 17 shows the RMSEP values of CH_4_, C_2_H_6_, and C_2_H_4_ for the models trained with different numbers of measured spectra. As expected, the RMSEP values decrease with the increase of the number of measured spectra. Therefore, more measured spectra should be selected to achieve high calculation accuracy. In practice, the number can be determined according to different accuracy requirements.

Now, we conclude three important trends for selecting measured spectra. One is the uniformly distribution in the concentration measurement range, the other is keeping samples as much as possible in the middle of the concentration range, and finally, trying to select more measured spectra.

#### 3.3.3. Simulation Interpolation Method

From the above measured and calculated results, it can be found that CH_4_ gas has the largest calculation error among the three gases. When CH_4_, C_2_H_6_, and C_2_H_4_ were mixed, the characteristic absorption of CH_4_ was completely overlapped with part of the characteristic absorption of C_2_H_6_ and C_2_H_4_, which resulted in the most severe influence on the absorption spectra of CH_4_.

**Figure 18:**
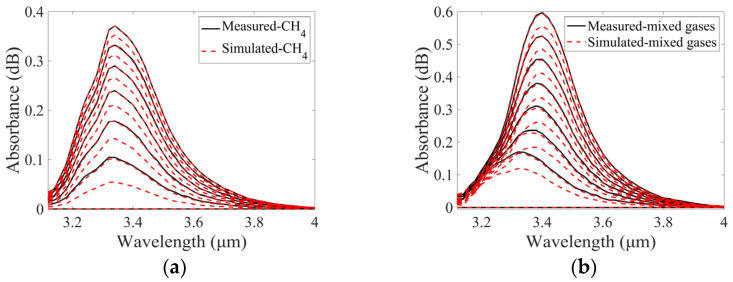
Comparison of measured and stimulated spectra of (**a**) CH_4_, (**b**) several mixed gases at different concentrations.

In order to minimize the error of CH_4_ between the calculated and actual concentrations, the simulation interpolation method was proposed for CH_4_. More simulated absorption spectra for CH_4_ were generated. The concentration interval for the CH_4_ simulated spectra was reduced from 210 ppm to 105 ppm, which means that the number of generated absorption spectra for CH_4_ was twice that of the other two gases. As shown in Figure 18, when the simulated concentration interval of CH_4_ is reduced, the number of simulated spectra for both CH_4_ and mixed gases is doubled. Consequently, the concentration interval of CH_4_ was 210/105/70/42 ppm, and the number of total simulated spectra for mixed gases was 343/637/931/1519, respectively.

**Figure 19:**
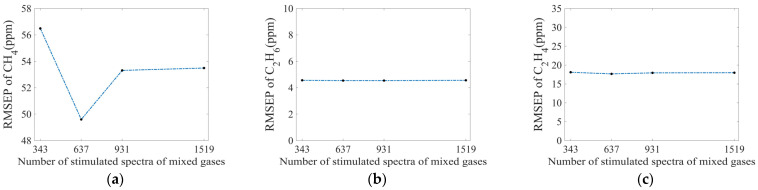
RMSEP values of (**a**) CH_4_, (**b**) C_2_H_6_, and (**c**) C_2_H_4_ obtained by training with different numbers of simulated spectra.

As shown in Figure 19a (with 27 measured spectra), as the number of simulated spectra of the mixed gases increases, the RMSEP value of CH_4_ first decreases and then increases, and finally tends to be constant. We conclude that selecting a smaller CH_4_ gas spectral interval can improve the calculation accuracy of CH_4_ gas. However, too small spectral intervals and too many simulated spectra, while increasing the error of simulated spectra, are not conducive to improving accuracy.

We note from Figure 19b,c that the RMSEP values for C_2_H_6_ and C_2_H_4_ are almost unchanged in this process. It shows that the simulation interpolation for CH_4_ has little effect on the error between calculated and actual concentrations for C_2_H_6_ and C_2_H_4_ gases.

**Figure 20:**
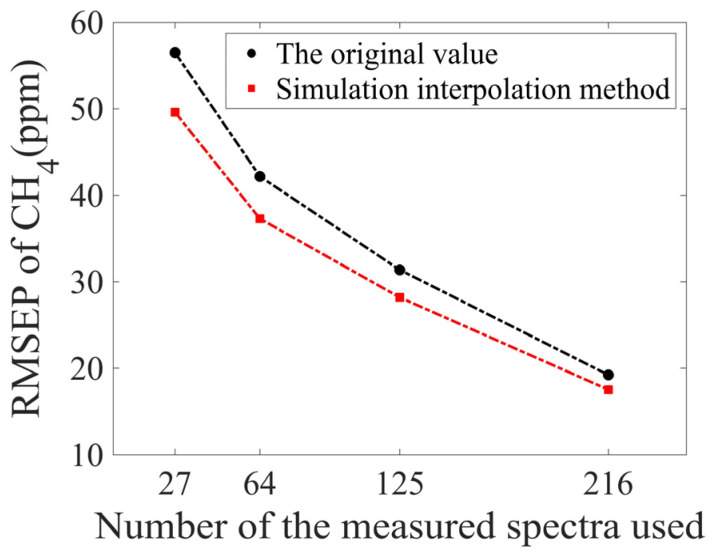
Comparison of RMSEP values of CH_4_ before and after using simulation interpolation method.

The performance of the simulation interpolation method for CH_4_ with more measured spectra was also investigated. It can be seen in Figure 20 that the RMSEP value of CH_4_ reduces with the increase of measured spectra from 27 to 216. And obviously, the calculation error with the interpolation method is smaller than that of the error without the method.

## 4. Conclusions

In this work, a CH_4_-C_2_H_6_-C_2_H_4_ multi-gas sensing system based on mid-infrared spectral absorption was proposed and established. The portable system utilized a commercially available blackbody radiation broadband light source and a tunable FP filter detector. The gas absorption cell is a flexible infrared waveguide inner coated with Ag and AgI optical film. It has low loss in the wavelength band of the intrinsic absorption of the measured gases, which improves the signal-to-noise ratio and achieves lower LODs. The LODs for single-component gases CH_4_, C_2_H_6_, and C_2_H_4_ were 7.33 ppm, 2.13 ppm, and 8.09 ppm, respectively.

Because the absorption spectra are highly overlapping for CH_4_, C_2_H_6_, and C_2_H_4_ mixed gases, the SVM algorithm was used to calculate the concentrations of each gas. A total of 343 absorption spectra were measured by the system for the gas mixture samples with various concentration combinations. The RMSEP values of CH_4_, C_2_H_6_, and C_2_H_4_ were 15.91 ppm (1.26%), 1.64 ppm (0.57%), and 6.95 ppm (0.55%), respectively.

In practice, system costs can be reduced by reducing the number of measured gas samples. Fewer measured spectra were used for spectra generation, and the SVM algorithm was trained by a large amount of generated spectra. The method of selecting measured spectra is important for calculation accuracy. Research results show three important trends for selecting measured spectra. One is the uniform distribution in the concentration measurement range, the other is keeping samples as much as possible in the middle of the concentration range, and finally, trying to use more measured spectra. Furthermore, a simulation interpolation method was proposed to enhance the accuracy for CH_4_ calculations.

The FP detector utilized in this work has a detection wavelength range of 3.1 to 4.4 μm. In the future, the integration of multiple FP detectors has the potential to expand the system’s detection wavelength range and enhance its capacity to detect diverse gas species. Infratec has launched four FP detectors that can detect different bands: 3.1–4.4 μm, 3.8–5.0 μm, 5.5–8.0 μm, and 8.0–10.5 μm (Infratec, LFP3144C-337, LFP3850C-337, LFP5580C-337, LFP80105C-337, Dresden, Germany). It is possible to integrate these four detectors to achieve measurements in the 3.1~10.5 μm wide band. The system needs to be improved accordingly during implementation. The fabrication of the HWGs needs to be carried out according to the different detection bands, and then the four HWGs and their corresponding detectors are integrated. Furthermore, the coupling joints between the light source and the gas cell as well as between the gas cell and the detectors have to be redesigned.

## Figures and Tables

**Table 2:** RMSEC and RMSEP values of CH_4_, C_2_H_6_, and C_2_H_4_ using SVM.

Gas	RMSEC	RMSEP
CH_4_	9.97 ppm (0.79%)	15.91 ppm (1.26%)
C_2_H_6_	0.36 ppm (0.13%)	1.64 ppm (0.57%)
C_2_H_4_	1.47 ppm (0.12%)	6.95 ppm (0.55%)

## Data Availability

The data presented in this study are available upon request from the corresponding author. The data are not publicly available due to privacy restrictions.

## Abbreviations

The following abbreviations are used in this manuscript:

FNE	Fluorescence noise elimination
FP	Fabry–Pérot
FRES	Fiber-enhanced Raman spectroscopy
HC-ARF	Hollow-core anti-resonant fiber
HWG	Hollow waveguide
IR	Infrared spectral absorption
LOD	Limit of detection
RBF	Radial basis function
RMSEC	Root mean square error of calibration
RMSEP	Root mean square error of prediction
SVM	Support vector machine
